# A cash-based intervention and the risk of acute malnutrition in children aged 6–59 months living in internally displaced persons camps in Mogadishu, Somalia: A non-randomised cluster trial

**DOI:** 10.1371/journal.pmed.1002684

**Published:** 2018-10-29

**Authors:** Carlos S. Grijalva-Eternod, Mohamed Jelle, Hassan Haghparast-Bidgoli, Tim Colbourn, Kate Golden, Sarah King, Cassy L. Cox, Joanna Morrison, Jolene Skordis-Worrall, Edward Fottrell, Andrew J. Seal

**Affiliations:** 1 Institute for Global Health, University College London, London, United Kingdom; 2 Concern Worldwide, Dublin, Ireland; 3 Norwegian Refugee Council, Nairobi, Kenya; 4 Concern Worldwide Somalia, Nairobi, Kenya; Epicentre, FRANCE

## Abstract

**Background:**

Somalia has been affected by conflict since 1991, with children aged <5 years presenting a high acute malnutrition prevalence. Cash-based interventions (CBIs) have been used in this context since 2011, despite sparse evidence of their nutritional impact. We aimed to understand whether a CBI would reduce acute malnutrition and its risk factors.

**Methods and findings:**

We implemented a non-randomised cluster trial in internally displaced person (IDP) camps, located in peri-urban Mogadishu, Somalia. Within 10 IDP camps (henceforth clusters) selected using a humanitarian vulnerability assessment, all households were targeted for the CBI. Ten additional clusters located adjacent to the intervention clusters were selected as controls. The CBI comprised a monthly unconditional cash transfer of US$84.00 for 5 months, a once-only distribution of a non-food-items kit, and the provision of piped water free of charge. The cash transfers started in May 2016. Cash recipients were female household representatives. In March and September 2016, from a cohort of randomly selected households in the intervention (*n* = 111) and control (*n* = 117) arms (household cohort), we collected household and individual level data from children aged 6–59 months (155 in the intervention and 177 in the control arms) and their mothers/primary carers, to measure known malnutrition risk factors. In addition, between June and November 2016, data to assess acute malnutrition incidence were collected monthly from a cohort of children aged 6–59 months, exhaustively sampled from the intervention (*n* = 759) and control (*n* = 1,379) arms (child cohort). Primary outcomes were the mean Child Dietary Diversity Score in the household cohort and the incidence of first episode of acute malnutrition in the child cohort, defined by a mid-upper arm circumference < 12.5 cm and/or oedema. Analyses were by intention-to-treat. For the household cohort we assessed differences-in-differences, for the child cohort we used Cox proportional hazards ratios. In the household cohort, the CBI appeared to increase the Child Dietary Diversity Score by 0.53 (95% CI 0.01; 1.05). In the child cohort, the acute malnutrition incidence rate (cases/100 child-months) was 0.77 (95% CI 0.70; 1.21) and 0.92 (95% CI 0.53; 1.14) in intervention and control arms, respectively. The CBI did not appear to reduce the risk of acute malnutrition: unadjusted hazard ratio 0.83 (95% CI 0.48; 1.42) and hazard ratio adjusted for age and sex 0.94 (95% CI 0.51; 1.74). The CBI appeared to increase the monthly household expenditure by US$29.60 (95% CI 3.51; 55.68), increase the household Food Consumption Score by 14.8 (95% CI 4.83; 24.8), and decrease the Reduced Coping Strategies Index by 11.6 (95% CI 17.5; 5.96). The study limitations were as follows: the study was not randomised, insecurity in the field limited the household cohort sample size and collection of other anthropometric measurements in the child cohort, the humanitarian vulnerability assessment data used to allocate the intervention were not available for analysis, food market data were not available to aid results interpretation, and the malnutrition incidence observed was lower than expected.

**Conclusions:**

The CBI appeared to improve beneficiaries’ wealth and food security but did not appear to reduce acute malnutrition risk in IDP camp children. Further studies are needed to assess whether changing this intervention, e.g., including specific nutritious foods or social and behaviour change communication, would improve its nutritional impact.

**Trial registration:**

ISRCTN Registy ISRCTN29521514.

## Abstract 2

Carlos S Grijalva-Eternod and colleagues present results for acute malnutrition, dietary diversity, and other nutrition-related outcomes from their non-randomised trial of a CBI for displaced families with young children.

## Author summary

**Why was this study done?:**

- Interventions that include the provision of cash have become ubiquitous within most humanitarian responses to crisis.
- The transfer of cash often aims to prevent children becoming acutely malnourished, but it is still unknown whether it works as a preventive intervention in different settings.
- Somalia has been affected by conflict since 1991, periodically forcing large-scale displacement and increasing mortality. Somalia has one of the highest levels of acute malnutrition observed in children aged less than 5 years.
- Evidence is needed to understand whether, in Somalia, a humanitarian intervention involving cash transfers would reduce the risk of children becoming malnourished and/or improve conditions that are often associated with malnutrition.

**What did the researchers do and find?:**

- We studied the changes in the risk of children aged 6–59 months becoming undernourished and the changes in the children’s dietary diversity following a cash-based humanitarian intervention in a population of internally displaced persons living in camps in peri-urban Mogadishu, Somalia.
- We compared the occurrence of acute malnutrition in all children aged 6–59 months living in the 10 camps that received the intervention with 10 camps that did not receive the intervention. We also compared the children’s dietary diversity in a sample of 240 households.
- Diet diversity appeared to improve in children from households receiving the cash-based humanitarian intervention. An apparent improvement in diet and food security was also observed in their mothers or primary carers and in their households.
- However, despite this apparent improvement in food security and diet diversity, we did not observe an associated reduction in the risk of children becoming acutely malnourished in camps receiving the cash-based humanitarian intervention.

**What do these findings mean?:**

- Humanitarian interventions involving cash transfers have consistently been shown to improve beneficiaries’ food security and diet. However, malnutrition has many causes, and this study provides evidence that these interventions are not always effective in preventing children becoming acutely malnourished.
- The study limitations include the fact that the intervention was allocated to camps based on vulnerability. This renders the negative finding for reducing malnutrition more difficult to interpret. In addition, we could not collect more information on undernutrition to better understand other potential changes, and the overall occurrence of acute malnutrition in the children was lower than we anticipated.
- It is unclear why this humanitarian intervention did not appear to reduce the risk of malnutrition in children. Future work is necessary to understand whether modifications to this intervention, such as adding specific nutritious foods or social and behaviour change communication, could positively affect its ability to prevent children from becoming acutely malnourished.

## Introduction

It is estimated that in low- and middle-income countries acute malnutrition affects about 52 million children aged <5 years [1]. Acute malnutrition prevalence and severity is usually greater in emergency-affected populations such as in natural disasters or conflicts [2]. This is a serious global health concern as acute malnutrition is a leading cause of childhood mortality, accounting for 11.5% of total deaths, and contributes significantly to the overall disease burden [3,4].

Since 1991, political instability and conflict, coupled with natural disasters such as droughts, have afflicted Somalia and have led to one of the highest global prevalences of acute child malnutrition (17.4% as of April–June 2017) [5–7]. This state of conflict has disrupted not only regional agriculture and trade, but also humanitarian access, forcing displacement and increasing mortality in this population [5,8,9]. Furthermore, trade disruption has exacerbated food insecurity due to increased food prices, especially in urban areas [9]. In 2017–2018, it is estimated that 1.2 million children in Somalia will be acutely malnourished, of which over 230,000 will be severely malnourished [7]. The group most affected by food insecurity and acute malnutrition is internally displaced persons (IDPs), who often live in camps in peri-urban areas, lacking access to essential basic services [7].

Within a humanitarian response, there are various interventions commonly used for the prevention of acute malnutrition [10]. Among these, cash-based interventions (CBIs) have recently gained popularity compared to conventional food-based interventions [11]. CBIs aim to improve the beneficiaries’ ability to acquire food and/or other needs, and they often have additional multi-sectoral objectives, such as enabling livelihood investments, and can improve health outcomes [12–14]. Furthermore, CBIs are perceived as a cost-effective nutritional intervention that improves beneficiary satisfaction and that has a positive impact on local economies [13].

CBIs were first implemented at scale in Somalia in 2011 to respond to the famine crisis and, given the existence of functioning markets in this context and the lack of humanitarian access to southern Somalia, were seen as an essential approach for providing food assistance [15]. However, evidence of nutritional impact of CBIs in this context is lacking.

Conducted by the Research on Food Assistance for Nutritional Impact (REFANI) consortium, the REFANI–Somalia study is 1 of 3 studies—the other 2 being in Niger and Pakistan [16,17]—that seek to contribute to the evidence base regarding the nutritional impact of humanitarian CBIs. Using a non-randomised cluster design, this study aimed to assess whether a CBI, allocated to beneficiaries following a vulnerability assessment, would improve child dietary diversity and reduce acute malnutrition risk in children aged 6–59 months living in IDP camps near Mogadishu, Somalia.

## Methods

### Ethics

The Ministry of Health and Human Services of the Federal Republic of Somalia (reference: MOH & HS /DGO/0469/August/2015) and the Research Ethics Committee of University College London (project ID: 1822/003) granted ethical approval. The study was registered on 19 January 2016 with ISRCTN (ISRCTN29521514).

Informed verbal consent was obtained from camp leaders in all IDP camps before starting data collection. In addition, following a detailed explanation of the study objectives and data collection process, informed verbal and written consent was obtained from caregivers in the participating households. Study participants were informed about their right to withdraw from the study and that participation in or withdrawal from the study would not affect their entitlement to humanitarian assistance. Confidentiality and the data security of the respondents were ensured by the removal of any information from datasets that could be used to identify respondents or their location.

### Study setting

The study was conducted in IDP camps located in Weydow area, Deyniile district, Mogadishu. Deyniile and Dharkenley, 2 of the 17 districts of Banaadir region, host the majority of IDPs in Mogadishu, who are primarily from marginalised tribes or minority groups [18]. Concern Worldwide has been implementing multi-sector development and humanitarian assistance programmes in Somalia since 1986.

The IDP camps in Weydow area are spontaneous and privately run settlements that are often overcrowded and lack basic sanitation and health services, and residents face recurrent evictions. Morbidity estimates (diarrhoea, pneumonia, and fever) in these IDP camps are high and are considered a major driver of high estimates of acute malnutrition [6]. During the wet seasons (*Gu* and *Deyr*, April–June and October–December, respectively), morbidity estimates, primarily from diarrhoea, increase [19]. Most of the IDPs were previously agro-pastoralists and riverine farmers who lived in the Bay, Bakool, and Shabelle regions [18]. Now, their primary livelihood sources are casual labour, petty trading, and humanitarian assistance received from local and international humanitarian organisations [19].

### Study design

Details of the study design have been published elsewhere [20]. The study was a non-randomised cluster trial, where IDP camps were the cluster units. Ten clusters were first selected for the CBI based on vulnerability criteria, following a routine needs assessment exercise conducted by Concern Worldwide. The routine needs assessment ranked vulnerability based on whether (1) households were headed by elderly people, children, women, or people with disabilities, (2) there were orphans in the household, (3) all household members had no income sources, (4) households had children that had recently recovered from acute malnutrition, (5) households had high dependency ratios, (6) households had pregnant and/or lactating women, and (7) the camp had been relocated recently following eviction from a previous location. In our study, a recent eviction was the primary criterion for camp selection. The intervention was not randomised in our study because the available funds were allocated based on need using the vulnerability criteria listed above. The number of clusters selected for intervention was determined by the availability of funds. All the households in the selected intervention clusters were registered for the CBI.

Afterwards, the control clusters were selected, which included 10 IDP camps located adjacent to the intervention clusters. These control clusters were not targeted to receive the intervention and were known to not have received CBIs recently. The control clusters contained an average of 236 households (range 70–433 households) and the intervention clusters had an average of 113 households (range 48–215 households).

### Intervention

The CBI took place in 2016 and comprised 3 elements: (1) a monthly unconditional cash transfer of US$84.00/month for 5 months, (2) a once-only distribution of a non-food-items kit, and (3) the provision of piped water free of charge, through tap stands.

The monthly cash amount was based on the cost of the Minimum Expenditure Basket developed by the Food and Agriculture Organization’s Food Security and Nutrition Analysis Unit–Somalia. The Minimum Expenditure Basket represented a minimum set of basic food items such as sorghum, vegetable oil, and sugar meeting the 2,100 kilocalories/person/day basic energy requirement for a household of 6–7 members, and non-food items such as water, kerosene, firewood, soap, and cereal grinding costs. Upon registration, a household female representative received a mobile phone SIM card with a unique number through which they received the cash transfer via mobile money transfer made by the contracted telecommunications company (Hormuud Telecom Somalia). The CBI targeted women as the household cash recipients on the assumption that their spending was more likely to benefit their children [21]. Cash transfers were done on May 30, June 25, July 25, August 25, and September 29, 2016.

The non-food-items kit comprised 1 plastic sheet, 2 mosquito nets, 1 blanket, 1 sleeping mat, 1 kitchen set, 1 bar of soap, 2 collapsible jerry cans, and 1 set of sanitary pads. The non-food-items kits were distributed in January and February 2016.

In addition to providing the CBI, Concern Worldwide provided support and equipment to the local maternal and child health and nutrition centres, where malnourished children and pregnant and lactating women in the study area could access treatment. In addition, Concern Worldwide and other non-governmental organisations supported building of improved pit latrines in most of the IDP camps included in both arms of the study.

### Study components and participants

The study consisted of 3 components for data collection: a household cohort, a child cohort, and a process evaluation.

#### Household cohort

This cohort was set up to evaluate the impact of the CBI on known malnutrition risk factors. The household cohort, comprising 12 households from each cluster (240 households in total), was surveyed March 13–29 before the cash transfers and September 1–25 after having received at least 3 of the monthly distributions of cash. Households were randomly selected from each cluster using standard nutrition survey methodology [22]. Briefly, a random direction was selected from the centre of a cluster; all households laying in that direction to the edge of the enumeration area were numbered, and 1 was selected at random. All remaining households were then sequentially selected by choosing the next-nearest household to the right. Only households with children aged <5 years were sampled. If a household was found not to have children aged <5 years, this household was skipped and the nearest one to the right was selected.

We collected data from these households, which included data for children aged <5 years and their mothers or primary carers. The data were gathered from household heads and mothers/primary carers regarding household demographics; wealth; food security; morbidity; access to healthcare; water, sanitation, and hygiene; and infant and young child feeding practices. We also collected anthropometric data from children aged 6–59 months to describe their nutritional status and acute malnutrition prevalence, but these data were not intended to be used to assess the impact of the CBI, given the household cohort’s limited sample size.

#### Child cohort

This cohort was set up to measure the monthly incidence of acute malnutrition. For the child cohort, 15 community health workers, grouped into 6 teams with 2 members each and 3 members as reserve, collected mid-upper arm circumference (MUAC) measurements and assessed bipedal pitting oedema (henceforth oedema) for all children aged 6–59 months, initially 2,337, representing an exhaustive sample of children living in all intervention and control clusters. This exhaustive sample of children in the child cohort included the children from the household cohort, although the data were not linked between these cohorts. The child cohort data were collected during household visits between June 21 and November 14. Children identified as acutely malnourished, i.e., with a MUAC < 12.5 cm and/or oedema, were referred to nutrition centres for treatment.

#### Process evaluation

A process evaluation was conducted to better understand the context in which the intervention was implemented, to document how the intervention was implemented compared to how it was planned, to explore mechanisms through which the intervention worked or failed to work, and to investigate any unexpected outcomes.

### Sample size

We performed sample size calculations using Stata (Release 14; StataCorp). To assess a change in malnutrition risk factors in the household cohort, we estimated a sample size able to detect a difference of 0.6 units in the mean Child Dietary Diversity Score (Child DDS). A positive change of about 2 units in Household Dietary Diversity Score (Household DDS) was observed in response to a CBI in Malawi [23], but other studies observed smaller changes [24,25]. Scarce data exist on Child DDS change following cash transfers, so we decided to power the study to detect a small change, assuming a baseline Child DDS of 3.0 with a SD of 1.5, a power of 80%, an alpha risk at 0.05, and an intra-cluster correlation coefficient of 0.01. The resulting sample size estimate was 120 children per arm. Based on previous nutrition survey data, we assumed an average of 1.2 children aged 6–59 months per household. Based on previous survey experience, we added 20% to allow for non-response. This resulted in a required total sample of 240 households.

To assess acute malnutrition incidence in the child cohort and detect a hazard ratio of 0.5, we assumed, based on operational guidance for caseload estimations [26], that the proportion of followed-up children who would develop a MUAC below 12.5 cm and/or oedema during a 6-month period was 7% and that there would be 20% loss to follow-up. Power was set at 80% and the alpha risk at 0.05. This resulted in a total sample size needed of 1,167 children, which equated to a rounded sample of 600 participants per study arm.

### Data collection and data handling

For the household cohort, we collected data using a structured questionnaire, translated into the local Somali language, on mobile devices running a proprietary version of Android Open Data Kit (PSI Mobile). The collected data comprised information at the household and individual level.

For the child cohort, we collected data using paper forms. These paper forms were later digitally captured, and data entry errors identified and corrected.

To ensure collection of high-quality data in the household and child cohorts, a 2-week training was implemented for enumerators and supervisors prior to the start of data collection. During this training, we piloted questionnaires. Details regarding key validation studies of the tools used, as well as information on their adaptation to the local context, are provided in S1 Table. All teams were supervised during field data collection. During home visits, if a child could not be found after repeated attempts, the reasons for absence from the household were ascertained from family or neighbours.

#### Household demographics

We obtained data on household demographic characteristics including the total number of members; members aged <5, 5–14, 15–49, 50–64, and ≥65 years; and the number of members living away. We calculated total, child, and aged dependency ratios as the number of members aged <15 years or aged ≥65 years, members aged <15 years, and members aged ≥65 years, respectively, divided by the number of members aged 15–64 years.

#### Water, sanitation, and hygiene

We obtained data via a questionnaire and observation on whether households had access to piped water, whether they needed to pay for this access, and the time that it took them to collect the water. We also obtained data on access to hand-washing facilities, soap availability, and the type of toilet. Households were classified as engaging in open defecation if members reported relieving themselves in open fields or if their household showed no evidence, reported or observed, of having access to a latrine.

#### Food security

We collected data to allow analysis of different food security indicators and domains. We applied a single 24-hour reported food recall to a checklist of 12 and 9 food groups to estimate Household DDS and Women Dietary Diversity Score (Women DDS), respectively, as recommended by the Food and Agriculture Organization of the United Nations [27]. The 24-hour dietary recall food groups for the household were (1) cereals, (2) white tubers and roots, (3) vegetables, (4) fruits, (5) meat, (6) eggs, (7) fish and seafood, (8) pulses, nuts, and seeds, (9) dairy products, (10) oils and fats, (11) sweets, and (12) spices, condiments, and beverages. The 24-hour dietary recall food groups for women were (1) all starchy staple foods, (2) beans, peas, nuts, and seeds, (3) dairy products, (4) flesh foods, (5) organ meat, (6) eggs, (7) vitamin-A-rich dark green leafy vegetables, (8) other vitamin-A-rich vegetables and fruits, and (9) other vegetables and other fruits. The potential score range for Household DDS and Women DDS was 0–12 and 0–9, respectively. Similarly, we applied a 24-hour reported food recall to a checklist of 7 food groups to estimate Child DDS as recommended by the World Health Organization [28]. The 24-hour food groups were (1) grains, roots, and tubers, (2) legumes and nuts, (3) dairy products, (4) flesh foods, (5) eggs, (6) vitamin-A-rich fruits and vegetables, and (7) other fruits and vegetables. The potential score range for the Child DDS was 0–7. In addition, we asked mothers/primary carers the number of meals consumed by the child in the past 24 hours.

The Food Consumption Score (FCS) is an indicator developed by the World Food Programme as a composite score based on dietary diversity, food frequency, and relative nutritional importance [29]. We applied a food frequency questionnaire for a 7-day recall period with a list of the same 12 food groups used for Household DDS, collecting the number of days that the household consumed each of the food groups. The data obtained were then condensed to 9 food groups: (1) main staples, (2) pulses, (3) vegetables, (4) fruits, (5) meat and fish, (6) dairy, (7) sugary products, (8) oils, and (9) condiments. As recommended, we multiplied the frequency of each of the 9 food groups by specific weighting values to obtain the overall score. The potential FCS range was 0–112. FCS values were categorised as poor (FCS = 0–21), borderline (FCS >21 but ≤35), and acceptable (FCS >35).

The Household Food Insecurity Access Scale (HFIAS) is an indicator used to distinguish food secure from insecure households [30]. The assumption that the experience of food insecurity causes predictable and quantifiable reactions and responses forms the basis of HFIAS. These reactions and responses include feelings of uncertainty or anxiety over food, perceptions that food is of insufficient quantity or quality, reported reduction of food intake and its consequences, and feelings of shame for resorting to socially unacceptable means to obtain food. The HFIAS tool consists of 9 questions that represent a generally increasing level of severity of food insecurity in the previous 4-week period the questions are answered by frequency of occurrence as never, rarely (1–2 times), sometimes (3–10 times), and often (>10 times), which are given a value of 0, 1, 2, and 3, respectively. The HFIAS score is the sum of the values for all 9 questions and ranges from 0 (food secure) to 27 (maximum food insecurity). Households were also grouped in HFIAS categories as food secure or mildly, moderately, or severely food insecure, as per recommendations [30].

The Household Hunger Scale (HHS) is a measure of household food deprivation [31]. The HHS is a derived indicator from HFIAS consisting of 3 of the 9 HFIAS questions that pertain to going to sleep hungry, having no food in the household because of a lack of resources, and passing a day and night without eating. Questions are answered by frequency of occurrence in the previous 4-week period as never, rarely or sometimes, and often; these answers are given a value of 0, 1, and 2, respectively. The HHS score is the sum of the values for these questions (range 0–6), and the household is then categorised as having little to no hunger (0–1), moderate hunger (2–3) or severe hunger (4–6).

The Reduced Coping Strategies Index (rCSI) is a simple tool applied in different contexts that assesses the frequency of days and the severity of the behavioural responses used by households when they cannot access enough food in a 7-day period [32]. The rCSI tool has 5 questions on coping strategies that include consuming less preferred foods, borrowing food, reducing meals, reducing portion sizes, and restricting adults’ food consumption to preserve children’s food consumption. As per recommendations, we weighted and summed the frequency responses to these questions to create an index where higher scores are indicative of greater food insecurity. The range obtained for the rCSI score was 0–56.

To obtain information on household food access on a longer timescale, we used the Months of Adequate Household Food Provisioning (MAHFP) indicator [33]. The MAHFP captures changes in the household’s ability to address vulnerability over a 12-month period. The household respondent was asked to recall which months in the past 12-month period the household did not have access to sufficient food to meet its needs. The number of months was summed, and this value was deducted from a value of 12.

#### Household expenditure and income

We collected 30-day and 4-month household expenditure for a list of 10 food groups and 27 non-food items. Values were standardised to a 30-day period, and the sum was used as a proxy for household expenditure. Household respondents were also asked to recall the past 30-day household income.

#### Child anthropometry and health

We measured, in duplicate, weight, length/height, and MUAC for children in the household cohort and MUAC for children in the child cohort, and obtained the mean. Weight was measured to 100-g precision using an electronic scale (SECA model 870). Length and height in children aged <24 months and ≥24 months, respectively, were measured to 1-mm precision using a stadiometer (Infant/Child/Adult ShorrBoard). MUAC was measured on the left arm to 1-mm precision using a TALC-UK insertion tape. Presence of oedema in children in both cohorts was recorded if an imprint remained in both feet after pressing them with the thumbs for 3 seconds. Children’s age in both cohorts was obtained using a calendar of events and was rounded to the nearest month. The anthropometric indices weight-for-length/height *z-*score (WHZ) and height-for-age *z-*score (HAZ) were calculated using the Stata *zanthro* command [34], and extreme values were flagged and excluded from analysis according to the cutoffs: mean WHZ ± 4 and mean HAZ ± 5 [35]. Acute malnutrition in children was defined as either a low MUAC (MUAC < 12.5 cm) and/or the presence of oedema (primary outcome definition) or a low WHZ (WHZ < −2) and/or the presence of oedema (secondary outcome definition). Stunting was defined as HAZ < −2. Mothers/primary carers were asked whether their children had been unwell in the last 4 weeks.

#### Process evaluation

For the process evaluation, we collected routine programme monitoring data and local health facility admissions and disease outbreak data to assess the implementation of the intervention and detect changes in the health and nutrition situation in the area. We monitored and recorded the provision of relief interventions by other non-governmental organisations and any significant developments in security, the economic situation, or infrastructure that may have influenced the health and nutrition situation of the IDP camp residents. We monitored delivery of the cash transfers to study participants by crosschecking the transfer records supplied by the telecommunications company with the list of study participants from the study database. This included (1) crosschecking alternative spellings of Somali names so that no recipient names were missed due to the way they were written, (2) harmonising the names of people who might have had different names recorded in the 2 databases, as it may happen that Somalis use either nicknames or their real names, and (3) identifying the names of people who might have used different names for the study and cash registration so as to double register to increase the resources that they get from humanitarian agencies. Native Somali speakers who knew the community members well, as they had worked in the same camps several months before the start of the cash distributions, performed the crosschecking. The study database had an exhaustive list of all children aged 6–59 months and their mothers or primary carers in the 20 clusters.

Qualitative data were collected from household members, community leaders, and health staff using interviews, focus group discussions, and observation notes. These data will be reported in a forthcoming publication.

### Primary and secondary outcomes

The primary outcome measures were (1) mean Child DDS values of children aged 6–59 months, assessed using data from the household cohort, and (2) incidence of acute malnutrition in children aged 6–59 months, as defined by a low MUAC and/or oedema, assessed using data from the child cohort.

The secondary outcome measures, assessed using data from the household cohort, were (1) prevalence of acute malnutrition in children aged 6–59 months, as defined by a low WHZ and/or oedema; (2) mean WHZ value in children aged 6–59 months; (3) mean 30-day household expenditure; (4) mean Household DDS; (5) mean FCS; (6) mean HFIAS score; (7) mean rCSI score; and (8) mean Women DDS.

### Data analysis

Data analysis was done using Stata. For the household cohort, prevalence and mean estimates at baseline and endline were computed using the survey (*svy*) command, which accounts for the clustering of values. We estimated the difference-in-differences (DiD) between the study arms as the arithmetic difference that resulted from subtracting the difference (endline minus baseline) in the control arm from the difference in the intervention arm. We tested, using a Student *t* test, whether the DiD was significantly different from 0 using the *lincom* command. To calculate confidence intervals for 0% or 100% proportions, we used the Newcombe–Wilson method without continuity correction [36]. We did not perform adjustment for multiple comparisons, but we opted to describe in full the statistical tests performed, as recommended [37].

As requested by *PLOS Medicine* reviewers, we also obtained the Child DDS DiD between the study arms using linear regression and ordered logistic regression, before and after adjustment for other variables that were found to be significantly different at baseline.

For the child cohort, we performed analysis of the incidence of acute malnutrition in the trial arms using Cox proportional hazards analysis. Survival time before the first episode of acute malnutrition was measured in days and calculated for everyone using the dates of household visits. When children were not found during a household visit, and were reported to be absent or dead, it was assumed that this reported status had existed for the entire period since the previous visit, and this time was not included in their calculated survival time. Measurements from children who died were treated as right censored data. The incidence of acute malnutrition was calculated using the *ir* command. The *stset* and *stcox* commands were used to define survival time and run the hazard model, and assumptions of proportionality were tested using the *estat phtest* command. For calculation of standard errors and statistical testing, the *svy linearized*: *stcox* command was used to account for clustering.

## Results

### Intervention implementation

Records obtained from the telecommunications company showed that cash transfers took place as scheduled, with 1,313 beneficiary households receiving around US$420.00 in total each. The amount received each month varied between US$83.00 and US$85.00 due to fluctuating market prices.

### Participant flow

**Fig 1:**
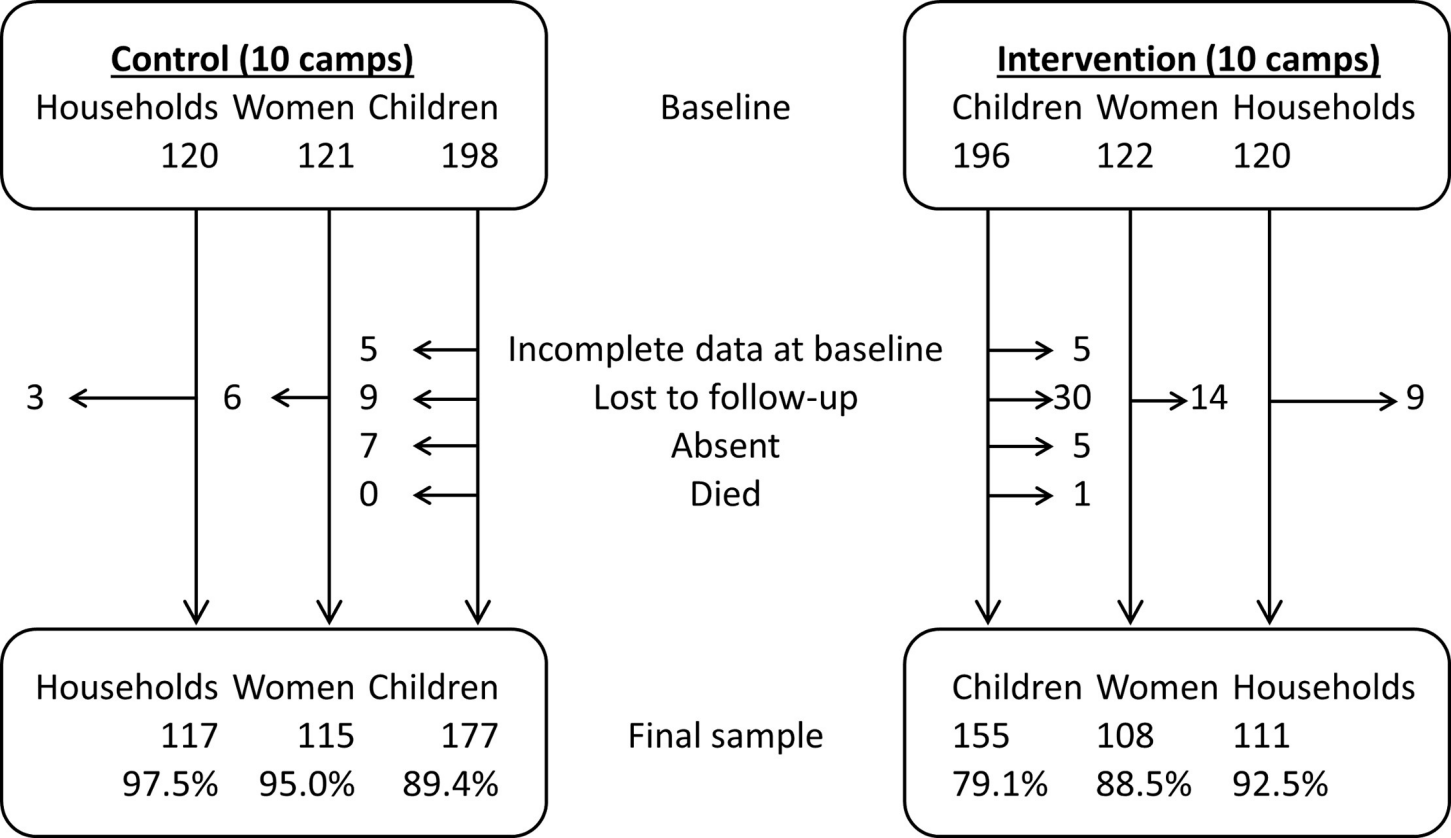
Household cohort participant flow diagram.

**Fig 2:**
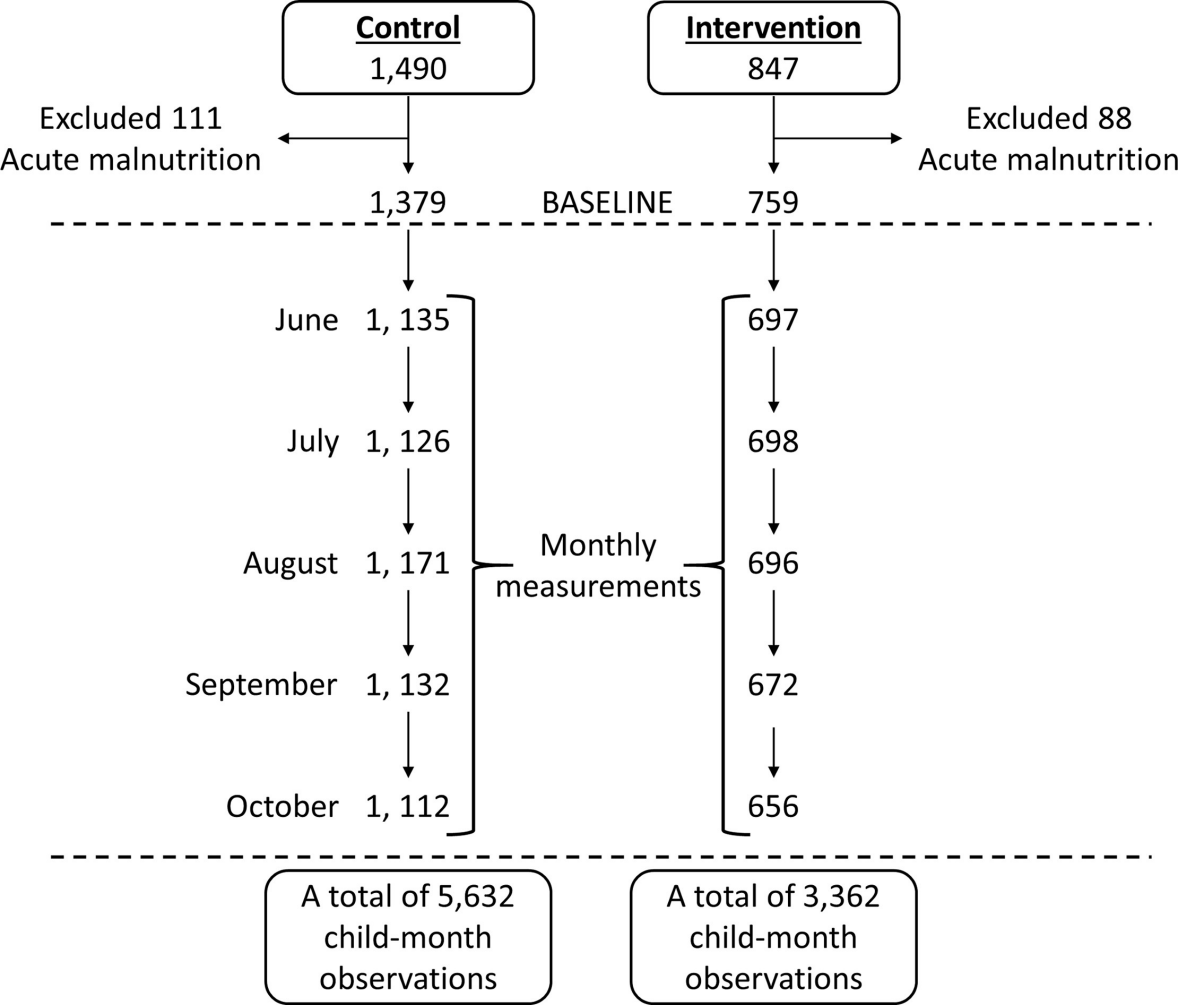
Child cohort participant flow diagram.

Fig 1 presents the participant flow of the household cohort. Overall, loss to follow-up at endline was 5%, 8%, and 16% for households, women, and children, respectively. Loss to follow-up was greater in the intervention arm. Fig 2 presents the participant flow of the child cohort. At baseline, 199/2,337 (8.5%) children presented with acute malnutrition and were therefore excluded from the analysis of incidence (111 [7.4%] and 88 [10.4%] from the control and intervention arms, respectively); oedema was not observed.

### Household cohort baseline characteristics

Table 1 shows the baseline characteristics. Overall, the households were reported to be mainly monogamous and female headed. Most households reported access to piped water that took them about an hour to collect, and few had hand-washing facilities in the household. Food utilisation indicators showed that households had a mean dietary diversity score above the mid-range value, and most had an acceptable FCS. However, food security indicators assessing the experience of food insecurity also showed that most households experienced severe food insecurity as most households engaged in coping strategies (S2 Table). Reported household expenditure was, on average, greater than reported income, with food accounting for a large proportion of that expenditure. The largest food expenditure item was cereals (S3 Table), whilst the largest non-food expenditures were on firewood and cooking fuel, drinking water, and transportation.

**Table 1:** Baseline characteristics of the household cohort.

Characteristic	Control		Intervention		Intervention minus control	
	Mean or percent	95% CI	Mean or percent	95% CI	Difference	95% CI
*Household*^1^						
Female head of household (%)	86.3	72.1; 93.9	96.4	89.3; 98.9	10.1	−1.2; 21.4
Head of household received Koranic education (%)	48.7	34.8; 62.8	36.9	19.1; 59.2	−11.8	−37.3; 13.8
Head of household received no other formal education (%)	90.8	86.0; 94.1	90.0	78.9; 95.6	−0.83	−9.69; 8.02
Polygamous arrangement (%)	10.0	3.51; 25.4	2.50	0.88; 6.92	−7.50	−17.9; 2.89
Total household members	5.89	5.25; 6.53	6.13	5.64; 6.62	0.24	−0.57; 1.04
Children aged <5 years	1.93	1.76; 2.11	1.81	1.54; 2.08	−0.12	−0.44; 0.20
Adolescents aged 5–14 years	2.03	1.56; 2.51	2.02	1.79; 2.25	−0.02	−0.54; 0.51
Adults aged 15–49 years	1.81	1.65; 1.97	2.02	1.85; 2.19	0.21	−0.03; 0.44
Women aged 15–49 years	1.01	0.94; 1.07	1.15	0.96; 1.35	0.14	−0.06; 0.35
Household members reported as away	1.09	0.17; 2.00	0.11	0.00; 0.22	−0.98**	−1.90; −0.05
Household total dependency ratio	2.40	2.13; 2.68	1.93	1.65; 2.21	−0.47*	−0.86; −0.08
Household child dependency ratio	2.38	2.11; 2.64	1.90	1.64; 2.16	−0.48*	−0.85; −0.11
Household aged dependency ratio	0.03	−0.01; 0.06	0.03	0.00; 0.07	0.01	−0.04; 0.06
Piped water in household (%)	99.0	93.7; 99.9	98.0	93.4; 99.5	−0.95	−3.91; 2.01
Household paid for water (%)	97.0	85.0; 99.3	100	96.7; 100	3.42	−1.90; 8.73
Hand-washing facility in household (%)	5.00	2.36; 10.8	2.00	0.46; 6.74	−3.33	−7.92; 1.27
Soap in household (%)	35.0	22.5; 50.0	32.0	17.5; 52.1	−2.61	−25.4; 20.2
Open defecation in household (%)	33.0	15.2; 58.2	11.0	2.78; 34.0	−22.5	−49.2; 4.14
Time to collect water (minutes)	59.5	41.3; 77.7	42.6	27.8; 57.3	−16.9	−40.4; 6.49
Months of Adequate Household Food Provisioning (MAHFP)	9.66	8.89; 10.4	10.3	9.67; 11.0	0.68	−0.34; 1.69
Meals in last 24 hours	1.79	1.70; 1.89	1.56	1.34; 1.78	−0.24*	−0.47; 0.00
Household Dietary Diversity Score (12 food groups)^2^	6.56	6.24; 6.87	7.16	6.84; 7.48	0.61*	0.16; 1.06
Food Consumption Score (FCS)^2^	52.1	47.2; 57.0	58.7	53.1; 64.2	6.54	−0.87; 14.0
Acceptable FCS (%)	79.0	0.66; 0.88	89.0	0.77; 0.95	9.7	−4.18; 23.6
Reduced Coping Strategy Index (rCSI) score^3^	28.8	26.4; 31.1	25.1	22.7; 27.5	−3.65*	−7.02; −0.28
Household Food Insecurity Access Scale (HFIAS) score^3^	17.4	16.2; 18.6	16.6	15.9; 17.3	−0.83	−2.19; 0.54
Severely food insecure (HFIAS) (%)	97.0	0.90; 0.99	97.0	0.89; 0.99	0.72	−4.84; 6.27
Household Hunger Scale (HHS) score^3^	2.65	2.14; 3.16	2.70	2.27; 3.14	0.05	−0.62; 0.72
Household having little to no hunger (HHS: 0–1) (%)	17.5	10.5; 27.8	13.3	4.90; 31.2	−4.30	−19.5; 10.9
Household income in last 30 days (US$)	55.3	50.0; 60.6	81.5	61.6; 101	26.2*	5.61; 46.8
Household expenditure in last 30 days (US$)	75.7	59.2; 92.2	92.1	69.3; 115	16.4	−11.8; 44.6
Household food expenditure in last 30 days (US$)	49.0	38.1; 59.9	68.8	51.7; 85.9	19.8	−0.51; 40.0
Household non-food expenditure in last 30 days (US$)	26.9	20.8; 32.4	23.1	16.5; 29.7	−3.46	−12.2; 5.32
Household expenditure/income ratio	1.64	1.04; 2.23	1.16	1.10; 1.23	−0.47	−1.07; 0.12
Food expenditure/household expenditure ratio	0.66	0.63; 0.69	0.76	0.73; 0.78	0.10**	0.06; 0.14
*Mother/primary carer*^1^						
Age (years)	28.4	26.8; 30.0	29.0	27.1; 30.9	0.62	−1.88; 3.13
Reported being pregnant (%)	20.0	12.3; 30.9	22.2	15.2; 31.3	2.22	−10.1; 14.5
Illiteracy (%)	94.8	84.4; 98.4	93.5	86.3; 97.1	−1.26	−9.08; 6.55
Did paid work last year (%)	10.4	4.17; 23.8	8.33	2.06; 28.2	−2.10	−16.6; 12.4
Age at first childbirth (years)	17.3	17.0; 17.7	17.4	16.8; 18.0	0.02	−0.75; 0.79
Women Dietary Diversity Score (9 food groups)	3.19	2.94; 3.45	3.58	3.32; 3.85	0.39*	0.03; 0.76
*Children*^1^ *(aged 6–59 months)*						
Age (months)	30.1	28.1; 32.1	32.4	30.0; 34.9	2.35	−0.79; 5.48
Male (%)	48.7	39.5; 57.9	48.8	41.5; 56.2	0.12	−11.8; 12.0
Health problem in last 30 days (%)	68.5	63.0; 73.6	74.2	62.2; 83.4	5.65	−6.27; 17.6
Measles vaccination (%)^4^	38.7	27.9; 50.7	64.3	41.5; 82.1	25.7*	1.32; 50.1
Length/height (cm)	82.8	81.5; 84.2	83.8	81.5; 86.0	0.95	−1.71; 3.60
Weight (kg)	10.7	10.4; 10.9	10.8	10.2; 11.4	0.13	−0.51; 0.76
Mid-upper arm circumference (MUAC, cm)	14.3	14.1; 14.6	14.1	13.7; 14.5	−0.18	−0.66; 0.30
Height-for-age *z-*score	−2.04	−2.26; −1.82	−2.29	−2.61; −1.97	−0.26	−0.64; 0.13
Weight-for-length/height *z-*score (WHZ)	−0.82	−1.06; −0.59	−0.83	−1.02; −0.64	−0.01	−0.31; 0.29
Acute malnutrition by low WHZ and/or oedema (%)	13.7	8.84; 20.7	14.9	10.5; 20.8	1.22	−6.53; 8.97
Acute malnutrition by low MUAC and/or oedema (%)	10.2	7.48; 13.7	12.3	5.22; 26.2	2.09	−8.40; 12.6
Stunting (%)	45.2	36.5; 54.2	55.5	44.6; 65.9	10.3	−3.78; 24.4
Child Dietary Diversity Score (7 food groups)^5^	2.47	2.27; 2.67	3.12	2.88; 3.35	0.65**	0.32; 0.98
Meals in last 24 hours	2.23	1.98; 2.47	1.79	1.69; 1.88	−0.44**	−0.71; −0.18

Acute malnutrition: Low WHZ: WHZ < −2. Low MUAC: MUAC < 12.5 cm.

^1^Households: control *n* = 117, intervention *n* = 111. Mothers/primary carers: control *n* = 115, intervention *n* = 108. Children: control *n* = 177, intervention *n* = 155.

^2^Higher is better.

^3^Higher is worse.

^4^Children aged 9–59 months.

^5^The intra-cluster correlation coefficient for Child Dietary Diversity Score was 0.12.

*p < 0.05

**p < 0.01.

At baseline, we found 3 main differences between the study arms (Table 1). First, the intervention arm appeared to be less poor and more food secure than the control arm. Households in the intervention arm reported greater income; spent proportionally more on food; presented greater dietary diversity scores for the household, women, and children; presented lower rCSI scores, and reported relying on less preferred and less expensive foods for a lower number of days (S2 Table). These households also reported spending more on meat, fish and eggs, dairy products, fats and oils, sugary products, and transportation (S3 Table). However, households and children in the intervention arm also reported consumption of fewer meals in the past 24 hours. Second, the intervention arm appeared to have more adults caring for dependants, as households in the intervention arm reported having fewer members away and presented lower household dependency ratios. Finally, the intervention arm reported a greater prevalence of measles vaccination. We did not observe other significant differences at baseline. S4 Table describes some of the baseline differences between arms associated to the vulnerability criteria used for targeting of the CBI. Unfortunately, the original data used for the humanitarian vulnerability assessment were not available for assessing differences at baseline.

### Household cohort Child Dietary Diversity Scores

Table 2 presents the differences observed between baseline and endline for each study arm and the DiD between these arms. We observed a significant increase in Child DDS of 0.53 (95% CI 0.01; 1.05). S5 Table presents the results of the linear regression and ordered logistic regression, requested by *PLOS Medicine* reviewers. A positive increase in Child DDS at endline was significant for the intervention in the unadjusted analysis in both linear and ordered logistic regression. This positive association continued after adjustment for other variables found to be significantly different at baseline along with child’s age.

**Table 2:** Difference between endline and baseline for each study arm and the difference-in-differences.

Characteristic	Endline minus baseline				Intervention minus control difference-in-differences	
	Control		Intervention			
	Difference	95% CI	Difference	95% CI	Difference	95% CI
*Household*^1^						
Follow-up period between baseline and endline (months)	5.51**	5.43; 5.60	5.52**	5.40; 5.64	0.01	−0.14; 0.15
Piped water in household (%)	−1.71	−6.04; 2.63	1.80	−0.60; 4.20	3.51	−1.44; 8.47
Household paid for water (%)	0.85	−6.22; 7.93	−98.2**	−100; −95.8	−99.1**	−106; 91.6
Hand-washing facility in household (%)	−4.27	−9.00; 0.45	5.41	−7.80; 18.6	9.68	−4.35; 23.7
Soap in household (%)	−18.8	−38.3; 0.65	30.6**	11.3; 50.0	49.4**	22.0; 76.9
Open defecation in household (%)	−12.0	−39.3; 15.3	−6.3	−20.0; 7.33	5.66	−24.8; 36.2
Time to collect water (minutes)	−30.9**	−51.5; −10.3	−28.1**	−43.7; −12.5	2.8	−23.1; 28.6
Months of Adequate Household Food Provisioning (MAHFP)	0.44	−0.93; 1.82	−0.36	−1.41; 0.69	−0.80	−2.53; 0.92
Meals in past 24 hours	0.03	−0.21; 0.28	0.82**	0.56; 1.08	0.79**	0.43; 1.14
Household Dietary Diversity Score (12 food groups)	0.57	−0.11; 1.26	1.57**	0.98; 2.16	0.99*	0.09; 1.90
Food Consumption Score (FCS)	10.9**	4.88; 16.9	25.7**	17.7; 33.6	14.8**	4.83; 24.8
Acceptable FCS (%)	12.0*	1.56; 22.4	10.8*	2.36; 19.3	−1.16	−14.6; 12.3
Reduced Coping Strategy Index (rCSI) score	−1.71	−4.30; 0.88	−13.3**	−18.3; −8.30	−11.6**	−17.2; −5.96
Household Food Insecurity Access Scale (HFIAS) score	0.15	−1.36; 1.65	−8.43**	−10.6; −6.31	−8.58**	−11.2; −5.97
Severely food insecure (HFIAS) (%)	1.71	−0.60; 4.02	−46.9**	−65.3; 28.4	−48.6**	−67.2; −29.9
Household Hunger Scale	0.15	−0.52; 0.83	−1.52**	−2.32; −0.72	−1.68**	−2.72; −0.63
Household having little to no hunger (HHS: 0–1) (%)	−9.04	−20.2; 2.09	44.0**	12.6; 75.4	53.0**	19.7; 86.3
Household income in last 30 days (US$)	−1.44	−8.82; 8.95	42.7**	23.0; 62.4	44.2**	23.1; 65.2
Household expenditure in last 30 days (US$)	−2.98	−17.4; 11.4	26.6*	4.87; 48.4	29.6*	3.51; 55.7
Household food expenditure in last 30 days (US$)	−2.89	−14.2; 8.42	7.90	−6.41; 22.2	10.8	−7.45; 29.0
Household non-food expenditure in last 30 days (US$)	−0.23	−5.39; 4.93	18.2**	7.40; 28.9	18.4**	6.46; 30.3
Household expenditure/income ratio	−0.23	−0.81; 0.35	−0.17**	−0.25; −0.09	0.06	−0.53; 0.65
Food expenditure/household expenditure ratio	−0.01	−0.05; 0.04	−0.10**	−0.16; −0.04	−0.09*	−0.17; −0.01
*Mother/primary carer*^1^						
Women Dietary Diversity Score (9 food groups)	0.47	−0.02; 0.96	1.84**	1.16; 2.52	1.37**	0.53; 2.21
*Children*^1^						
Health problem in last 30 days (%)	−12.4*	−24.1; −0.65	−16.8	−38.1; 4.59	−4.41	−28.8; 19.9
Length/height (cm)	3.03**	2.62; 3.43	3.04**	2.53; 3.55	0.01	−0.64; 0.66
Weight (kg)	0.96**	0.81; 1.11	0.85**	0.63; 1.08	−0.11	−0.38; 0.16
Mid-upper arm circumference (MUAC, cm)	0.42**	0.20; 0.64	0.35**	0.13; 0.57	−0.07	−0.38; 0.24
Height-for-age *z-*score	−0.16**	−0.24; −0.09	−0.07	−0.21; 0.07	0.10	−0.07; 0.26
Weight-for-length/height *z-*score (WHZ)	0.47**	0.37; 0.57	0.25*	0.03; 0.47	−0.22	−0.46; 0.03
Acute malnutrition by low WHZ and/or oedema (%)	−6.29*	−11.8; −0.78	−5.19*	−9.07; −1.32	1.09	−5.64; 7.83
Acute malnutrition by low MUAC and/or oedema (%)	−5.08*	−9.15; −1.02	−7.10*	−13.5; −0.72	−2.01	−9.58; 5.56
Stunting (%)	3.39	−0.18; 6.96	3.23	−3.32; 9.77	−0.16	−7.61; 7.29
Child Dietary Diversity Score (7 food groups)	0.47**	0.37; 0.57	1.03**	0.58; 1.47	0.53*	0.01; 1.05
Meals in past 24 hours	−0.06	−0.39; 0.28	0.87**	0.73; 1.00	0.92**	0.56; 1.28

Acute malnutrition: Low WHZ: WHZ < −2. Low MUAC: MUAC < 12.5 cm.

^1^Households: control *n* = 117, intervention *n* = 111. Mothers/primary carers: control *n* = 115, intervention *n* = 108. Children: control *n* = 177, intervention *n* = 155.

*p < 0.05

**p < 0.01.

### Child cohort acute malnutrition incidence

Details of the child cohort at each round of measurement are presented in S6 Table. Data on the incidence of acute malnutrition and mortality are shown in Table 3. Overall, the incidence of acute malnutrition and mortality appeared lower in the intervention arm, but the unadjusted (0.83, 95% CI 0.48; 1.42) and adjusted hazard ratios (0.94, 95% CI 0.51; 1.74) were not significant, indicating no protective effect of the intervention. Forty-six children (2.2%) received hospital treatment, with no difference between study arms.

**Table 3:** Effect of the intervention on time to first episode of acute malnutrition or death.

Variable	Control	Intervention	*p*-Value
Sample, *n*	1,379	759	
Child-months observed, *n*	5,632	3,362	
Acute malnutrition, *n*	52	26	
Oedema, *n*	0	0	
Hospital treatment, *n*	30	16	
Death, *n*	11	3	
Acute malnutrition incidence rate, cases/100 child-months	0.92 (0.53; 1.14)	0.77 (0.70; 1.21)	
Mortality rate, deaths/100 child-months	0.20 (0.11; 0.35)	0.09 (0.03; 0.28)	
Acute malnutrition incidence hazard ratio	Reference	0.83 (0.48; 1.42)	0.5
Adjusted^1^ acute malnutrition incidence hazard ratio	Reference	0.94 (0.51; 1.74)	0.8
Mortality hazard ratio	Reference	0.47 (0.12; 2.22)	0.3

Acute malnutrition was defined as mid-upper arm circumference < 12.5 cm and/or oedema. Values in parentheses are 95% CIs.

^1^Adjusted for age (in months) and male sex. Only age was significant (hazard ratio 0.90, 95% CI 0.88; 0.93; *p <* 0.01).

In addition to the intention-to-treat analysis, we analysed the incidence data including only the 61.3% of children in the intervention clusters whose mother or primary carer had been confirmed as a recipient of the cash transfer using the transfer list from the telecommunications company. The analysis yielded similar results (see S7 Table).

### Household cohort difference-in-differences

In Table 2 we observed 3 patterns of change associated with the intervention, as indicated by a significant DiD. First, water, sanitation, and hygiene indicators improved, with an elimination of payment for water and an increase in soap presence in households. Second, household wealth improved, with significant increases in household income, total expenditure, and non-food expenditure, but without an increase in food expenditure. Lastly, food security improved, with increases in most household food security indicators, dietary diversity for women, and meal frequency for children. No significant DiD values were observed for children’s anthropometric data, although both arms showed significant improvements. In addition, we observed a reduction of reported health problems in children that was significant only in the control arm, but with no significant DiD between arms. Interestingly, the improvements in dietary diversity appeared to be higher in women than in children, although this difference was not tested. The DiD values for dietary diversity for households, women, and children represented a relative increase from baseline values in the intervention arm of 14%, 38% and 17%, respectively.

### Household cohort coping strategies

Table 4 presents details on the 5 coping strategies making up the rCSI. The proportion of households that employed each of the 5 coping strategies was significantly reduced in the intervention arm compared to the control arm. Similarly, the average number of days each coping strategy was employed was significantly decreased among those receiving the intervention, compared to the control arm, for most coping strategies, except for reliance on less preferred and less expensive foods. Of the 5 coping strategies investigated, adults’ restricting food consumption in order for small children to eat and reducing the number of meals eaten in a day showed the largest decreases in the intervention arm.

**Table 4:** Difference between endline and baseline for each study arm and the difference-in-differences for coping strategies.

Coping strategy	Endline minus baseline				Intervention minus control difference-in-differences	
	Control^1^		Intervention^1^			
	Difference	95% CI	Difference	95% CI	Difference	95% CI
*Number of days using strategy/7 days*						
Relied on less preferred and less expensive foods	−1.04*	−2.06; −0.03	−1.19**	−1.86; −0.52	−0.15	−1.36; 1.07
Borrowed food or relied on help from friends/family	−0.01	−0.78; 0.77	−1.59**	−2.37; −0.80	−1.58**	−2.68; −0.48
Reduced number of meals eaten in a day	−0.06	−0.74; 0.62	−1.82**	−2.57; −1.07	−1.76**	−2.77; −0.75
Limited portion size at mealtimes	−0.23	−0.75; 0.29	−1.79**	−2.51; −1.08	−1.56**	−2.45; −0.67
Restricted consumption by adults in order for small children to eat	0.10	−0.67; 0.87	−1.73**	−2.45; −1.01	−1.83**	−2.89; −0.78
*Percent of households using strategy/7 days*						
Relied on less preferred and less expensive foods	0.00	0.00; 0.03	−16.2**	−24.8; −7.61	−16.2**	−24.8; −7.61
Borrowed food or relied on help from friends/family	−2.56	−7.81; 2.68	−27.9**	−46.7; −9.16	−25.4*	−44.9; −5.87
Reduced number of meals eaten in a day	1.71	−3.34; 6.76	−39.6**	−58.3; −20.9	−41.3**	−60.7; −22.0
Limited portion size at mealtimes	1.71	−1.76; 5.18	−32.4**	−46.6; −18.3	−34.1**	−48.7; −19.5
Restricted consumption by adults in order for small children to eat	5.98	−9.19; 21.2	−52.3**	−71.0; −33.5	−58.2**	−82.3; −34.1

^1^Control *n* = 117, intervention *n* = 111.

*p < 0.05

**p < 0.01.

### Household cohort household expenditure

Table 5 presents the DiD values between the trial arms for 30-day household expenditures for food and non-food items. Overall, the results show that households receiving the intervention significantly increased their expenditure, primarily on non-food items. Regarding food items, households receiving the intervention significantly increased their expenditure only on dairy products. Households increased their non-food expenditure on cooking fuel, health, clothing, debt repayments, and housing, whilst significantly decreasing their expenditure on drinking water, presumably due to the inclusion of piped water free of charge in the intervention. The greatest increases in expenditure were for clothing and debt repayments.

**Table 5:** Difference between endline and baseline for each study arm and the difference-in-differences for 30-day household expenditure (in US dollars).

Item	Endline minus baseline				Intervention minus control difference-in-differences	
	Control^1^		Intervention^1^			
	Difference	95% CI	Difference	95% CI	Difference	95% CI
*Food*						
Cereals	−1.91	−6.25; 2.44	1.00	−8.90; 10.9	2.91	−7.91; 13.7
Roots and tubers	−0.34	−1.16; 0.47	0.53	−0.65; 1.71	0.87	−0.56; 2.31
Pulses, beans, and nuts	0.13	−1.01; 1.27	0.05	−1.40; 1.50	−0.07	−1.92; 1.77
Vegetables	0.44	−1.25; 2.12	1.12	−2.81; 5.04	0.68	−3.59; 4.95
Fruits	−1.48**	−2.18; −0.78	−1.31*	−2.30; −0.31	0.17	−1.04; 1.39
Meat, fish, and eggs	0.76	−0.40; 1.93	2.24*	0.09; 4.40	1.48	−0.97; 3.93
Dairy products	0.69	−1.16; 2.55	3.26**	1.74; 4.78	2.57*	0.17; 4.97
Fats and oils	−0.16	−1.08; 0.75	−0.11	−1.00; 0.78	0.05	−1.22; 1.33
Sugary products	−0.27	−1.41; 0.87	1.17	−0.47; 2.81	1.44	−0.55; 3.44
Condiments	−0.74**	−1.15; −0.34	−0.06	−0.76; 0.63	0.68	−0.13; 1.49
*Total food expenditure*	−2.89	−14.2; 8.42	7.90	−6.41; 22.2	10.8	−7.45; 29.0
*Non-food*						
Firewood/cooking fuel	−0.94	−2.13; 0.25	1.91	0.00; 3.82	2.85*	0.60; 9.31
Cigarettes, tobacco, khat	1.32	−0.55; 3.18	1.44*	0.13; 2.76	0.13	−2.16; 4.52
Drinking water	−0.67	−1.62; 0.29	−6.14**	−7.12; −5.15	−5.47**	−6.84; 0.27
Education	−0.18	−1.30; 0.95	0.93	−0.74; 2.59	1.10	−0.90; 3.11
Health	−0.96*	−1.76; −0.15	1.36	−0.45; 3.16	2.31*	0.34; 4.28
Clothing	2.82**	1.68; 3.96	7.69**	6.04; 9.33	4.87**	2.87; 6.87
Transport	−0.18	−2.05; 1.69	2.51	−0.94; 5.96	2.69	−1.24; 6.62
Debt repayment	0.22	−0.22; 0.66	4.92**	3.00; 6.85	4.70**	2.73; 6.68
Sending remittances	−0.06	−0.29; 0.17	1.11	−0.21; 2.43	1.17	−0.17; 2.51
Housing	−1.24	−3.19; 0.71	2.23**	0.78; 3.68	3.47**	1.04; 5.90
Shop or trading facilities	−0.10	−0.29; 0.10	0.38	−0.29; 1.06	0.48	−0.22; 1.18
Purchasing land	0.00	0.00; 0.03	0.00	0.00; 0.03	0.00	−0.03; 0.03
Farming items	0.01	−0.01; 0.03	0.08	−0.11; 0.27	0.07	−0.12; 0.26
Livestock	0.00	0.00; 0.03	0.26	−0.17; 0.70	0.26	−0.17; 0.70
Celebrations	−0.14*	−0.26; −0.02	0.03	−0.17; 0.23	0.17	−0.06; 0.40
*Total non-food expenditure*	−0.23	−5.39; 4.93	18.2**	7.40; 28.9	18.4**	6.46; 30.3

^1^Control *n* = 117, intervention *n* = 111.

*p < 0.05

**p < 0.01

## Discussion

### Summary of results

To our knowledge, this is the first study evaluating the nutritional impact associated with a humanitarian CBI on IDP children in Somalia. We found that after households received this CBI, household wealth and food security and children’s dietary diversity appeared to improve significantly. However, despite these improvements, we did not find evidence that this intervention was associated with an improvement in children’s nutritional status or with a reduced risk of developing acute malnutrition.

The impact of humanitarian CBIs on the nutritional status of children has also been studied, with mixed results, in other contexts, including Niger [17,38,39], Pakistan [40], Burkina Faso [41,42], and Congo [43]. However, direct comparisons are difficult because of contextual differences in the humanitarian emergency (e.g., seasonal hunger versus forced displacement); the modality, timing, and duration of the interventions; and the public health, livelihoods, and market environment in each study setting.

### Food security improvements

Improvement of household wealth and food security has often been observed following CBIs in different settings [44], as observed in our study and other humanitarian settings. Compared to control households that did not receive cash transfers, households receiving cash in other studies significantly increased their individual dietary diversity and reported increased consumption of nutritious food, animal protein, and iron-rich foods [40,41]. Similarly, in our study we observed apparent increases in household and individual dietary diversity (in mothers/primary carers and children) and increases in the reported consumption of more nutritious food groups such as dairy products. Furthermore, we observed changes across different food security indicators that reflected improvements in food utilisation and the experience of greater food security in the intervention arm. Interestingly, the increases in dietary diversity observed suggested that cash might have increased dietary diversity more among mothers/primary carers than among children. Although we lacked a large enough sample size to be able to assess further the relationship between the dietary diversity changes of women and children, this observed difference agrees with other changes in the reported household coping strategies. That is, the greatest change in coping strategies observed in households receiving the CBI was reduction in the restriction of food consumption by adults in order for children to eat, suggesting the possibility that children’s access to food was already prioritised in households prior to receiving the CBI.

### The CBI did not appear to reduce the risk of acute malnutrition

Our finding that a humanitarian CBI appeared to increase food security but was not associated with a reduced risk of developing acute malnutrition in children was also observed in a study in Burkina Faso and in one of the intervention arms in a study in Pakistan [40,42]. Conversely, improvements in the nutritional status of children were observed in a study in Congo and in another intervention arm in Pakistan study [40,43]. Among the possible reasons for the lack of nutritional impact in our study are that the cash transfer might not have been sufficiently large, that the cash transfer may not have been used in the most nutritionally optimal way because of potentially limited market choices, and that a positive nutritional impact might be observable only among those who are nutritionally vulnerable.

In the abovementioned study in Pakistan, a reduced prevalence of acute malnutrition was observed only in the arm receiving double the amount of money that the standard CBI recipient received (US$28.00 versus US$14.00 per month); the amounts were designed to cover 20% and 10% of the energy requirements of a typical household, respectively [16]. However, in our study the cash transfer was designed to cover 100% of the energy requirements of the typical household [20], but despite this relatively larger transfer, we observed no associated impact on nutritional status.

Targeting of cash transfers to individuals who are nutritionally vulnerable or providing specific nutritious foods together with cash transfers has been shown to reduce the risk of acute malnutrition in children. In Niger, children of families that received specific nutritious foods plus cash transfers showed a lower risk of acute malnutrition compared to those receiving cash transfers only [39]. Authors of that study suggested that this combination ensured that vulnerable children would consume foods that are more nutritious, as the risk of sharing the specific nutritious foods with other household members would be reduced compared to if the nutritious foods were provided without the cash transfer. In Congo, children affected by severe acute malnutrition showed a lower incidence of relapse when receiving cash transfers during outpatient therapeutic programme care and after discharge, for a total of up to 6 months [43]. In our study, no additional specific nutritious food was provided, and the additional components of the CBI were designed to improve water access and shelter infrastructure (plastic sheets) and to reduce the likelihood of malaria transmission (mosquito nets). Whether the addition of specific nutritious foods to the CBI, or providing social and behaviour change communication messages to influence expenditure decisions, could result in a lower acute malnutrition risk for children in this setting will need to be explored in future studies. Similarly, whether changing the way vulnerable households are selected for a CBI intervention in this context, such as using severe acute malnutrition cases as a primary household vulnerability criterion, might result in greater nutritional impact, although the perverse incentive potential of this criterion requires further assessment.

How CBIs in humanitarian settings might affect disease burden remains to be understood. In Pakistan, households in the intervention arms receiving cash transfers (the standard and the double cash) saw a significant reduction in reported fever/malaria compared to the control group (no cash), but only the arm receiving double the amount of money saw a significant reduction of reported respiratory infections. In contrast, in our study we observed a significant reduction in reported health problems in both arms, but no differences between these arms, despite clear evidence of a significant improvement of soap availability and the removal of the economic costs of accessing piped water in households receiving the CBI.

It should be noted that data collection for this study ended a few months before the onset of the 2017 Somalia drought and health emergency. Although evidence of this humanitarian emergency was evident as early as November 2016, large increases in acute malnutrition and mortality did not materialise until early 2017 [5,8]. Therefore, our findings represent the apparent impact of a humanitarian CBI on a chronically vulnerable, relatively stable population, rather than on a population undergoing an acute shock. The generalisability of our findings to other humanitarian contexts therefore needs to be carefully considered, as it is likely that a CBI would yield different results during periods of higher nutritional stress, such as during a drought.

### Strengths and limitations

There are limitations to our study, associated with the conflict and insecurity in this context, which made reducing risks to the study team and the beneficiary population paramount. First, the intervention was not randomised because it was allocated based on need, reducing our ability to guard against allocation bias and confounding. In addition, the data from the humanitarian vulnerability assessment used to allocate the intervention was not available for analysis. However, our results indicate that although the intervention group appeared to be wealthier and more food secure, most indicators suggested good matching between intervention and control groups. Second, to minimise the field presence of data collection teams, we recruited and followed a small cohort of households to assess food security indicators, but the sample was not adequate to assess nutritional impact or to study the relationship between changes in nutrition status and food security. This small sample also prevented us from assessing the relationship between morbidity and nutritional status and from collecting data on other important factors such as food preparation hygiene. Nonetheless, the large improvement in household food security indicators observed in the intervention group during this brief period provides a strong indication that, in this context, improving food security alone might be insufficient to improve the nutritional status of children. Third, due to logistics and security considerations, for the child cohort we did not collect other anthropometric measurements apart from MUAC. This prevented us from assessing the impact of the intervention on acute malnutrition, as defined by WHZ or stunting. Fourth, we did not collect information on the types of nutritious foods that were in the local markets, which could have affected the likelihood of the nutritional status of children being improved via cash transfers. Lastly, the observed acute malnutrition incidence was lower than expected (3.6% observed versus 7% expected), reducing our ability to detect significant differences.

There are also strengths to this study. To our knowledge, this is the first study to assess the associated nutritional impact of this type of humanitarian intervention in a population exposed to conflict and recent displacement. Other studies assessing the impact of humanitarian CBIs on nutritional outcomes have been undertaken on populations exposed to a seasonal hunger gap [17,38,39,41,42]. Our study provides evidence on what changes could be observed in populations exposed to conflict and recent displacement. In addition, this study used a varied selection of food security indicators, which allowed for a wider understanding of possible changes and aid interpretation. That most indicators in the intervention arm changed in a similar fashion provided us with greater confidence of the veracity of our findings.

## Conclusions

To conclude, our study showed that in this non-randomised trial, a humanitarian CBI appeared to increase household wealth and food security. However, the CBI did not appear to show an effect on the risk of developing acute malnutrition in children living in IDP camps. Future work is needed to understand whether modifications to this CBI—such as inclusion of additional components (e.g., provision of specific nutritious foods), changes in the targeting criteria, or social and behaviour change communication—could positively affect its ability to reduce malnutrition in this context. Furthermore, whether CBIs would reduce malnutrition risk in this context when a drought or other shock exacerbates the underlying food scarcity needs to be studied.

## Supporting information

S1 STROBE Checklist (PDF)

S1 Dataset The REFANI–Somalia household cohort. (ZIP)

S2 Dataset The REFANI–Somalia child cohort. (XLSX)

S1 Table Key validation studies and adaptation of tools. (PDF)

S2 Table Baseline coping strategies of the household cohort. (PDF)

S3 Table Baseline 30-day expenditure of the household cohort (in US dollars). (PDF)

S4 Table Baseline differences in indicators associated with the assessment of vulnerability and targeting of the intervention. (PDF)

S5 Table Linear and ordered logistic regression on endline Child Dietary Diversity Score. (PDF)

S6 Table Sample characteristics and MUAC at each surveillance time point. (PDF)

S7 Table Effect of the intervention on time to first episode of acute malnutrition or death. The intervention sample includes children whose mothers or primary carers were confirmed cash recipients.
(PDF)

## Abbreviations

CBI: cash-based intervention
Child DDS: Child Dietary Diversity Score
DiD: difference-in-differences
FCS: Food Consumption Score
HAZ: height-for-age *z-*score
HFIAS: Household Food Insecurity Access Scale
HHS: Household Hunger Scale
Household DDS: Household Dietary Diversity Score
IDP: internally displaced person
MAHFP: Months of Adequate Household Food Provisioning
MUAC: mid-upper arm circumference
rCSI: Reduced Coping Strategies Index
REFANI: Research on Food Assistance for Nutritional Impact
WHZ: weight-for-length/height *z-*score
Women DDS: Women Dietary Diversity Score
