# Supplementary material for: A cash-based intervention and the risk of acute malnutrition in children aged 6–59 months living in internally displaced persons camps in Mogadishu, Somalia: A non-randomised cluster trial
Source: PLoS Med. 2018 Oct 29;15(10):e1002684. doi: 10.1371/journal.pmed.1002684 (PMC6205571; doi:10.1371/journal.pmed.1002684)
Supplement: S1 STROBE Checklist — (PDF) [file pmed.1002684.s001.pdf]

STROBE Statement—Checklist of items that should be included in reports of ***cohort studies***

	Item No	Recommendation
**Title and abstract**	1	(*a*) Indicate the study's design with a commonly used term in the title or the abstract **Title page**
		(*b*) Provide in the abstract an informative and balanced summary of what was done and what was found **Abstract**
**Introduction**		
Background/rationale	2	Explain the scientific background and rationale for the investigation being reported **Introduction section, 3rd paragraph.**
Objectives	3	State specific objectives, including any pre-specified hypotheses **Introduction section, 5th paragraph.**
**Methods**		
Study design	4	Present key elements of study design early in the paper **Methods section, study design sub-section.**
Setting	5	Describe the setting, locations, and relevant dates, including periods of recruitment, exposure, follow-up, and data collection **Methods section, study design and study components and participants sub-sections.**
Participants	6	(*a*) Give the eligibility criteria, and the sources and methods of selection of participants. Describe methods of follow-up **Methods section, study components and participants sub-section.**
		(*b*) For matched studies, give matching criteria and number of exposed and unexposed **Methods section, study design sub-section.**
Variables	7	Clearly define all outcomes, exposures, predictors, potential confounders, and effect modifiers. Give diagnostic criteria, if applicable **Methods section, data collection and data handling sub-section.**
Data sources/ measurement	8*	For each variable of interest, give sources of data and details of methods of assessment (measurement). Describe comparability of assessment methods if there is more than one group **Methods section, data collection and data handling and primary and secondary outcomes sub-sections.**
Bias	9	Describe any efforts to address potential sources of bias **Methods section, study design sub-section, second paragraph, and data collection and data handling section, 3rd paragraph.**
Study size	10	Explain how the study size was arrived at **Methods section, sample size sub-section.**
Quantitative variables	11	Explain how quantitative variables were handled in the analyses. If applicable, describe which groupings were chosen and why **Methods section, data collection and data handling sub-section.**
Statistical methods	12	(*a*) Describe all statistical methods, including those used to control for confounding
		(*b*) Describe any methods used to examine subgroups and interactions
		(*c*) Explain how missing data were addressed
		(*d*) If applicable, explain how loss to follow-up was addressed
		(*e*) Describe any sensitivity analyses **Methods section, data analysis sub-section.**

**Results**		
Participants	13*	(a) Report numbers of individuals at each stage of study—eg numbers potentially eligible, examined for eligibility, confirmed eligible, included in the study, completing follow-up, and analysed
		(b) Give reasons for non-participation at each stage
		(c) Consider use of a flow diagram<br>**Results section, participant flow sub-section.**
Descriptive data	14*	(a) Give characteristics of study participants (eg demographic, clinical, social) and information on exposures and potential confounders
		(b) Indicate number of participants with missing data for each variable of interest
		(c) Summarise follow-up time (e.g., average and total amount)<br>**Tables 1 & 2**
Outcome data	15*	Report numbers of outcome events or summary measures over time<br>**Tables 2 & S6 Table**
Main results	16	(*a*) Give unadjusted estimates and, if applicable, confounder-adjusted estimates and their precision (e.g., 95% confidence interval). Make clear which confounders were adjusted for and why they were included
		(*b*) Report category boundaries when continuous variables were categorized
		(*c*) If relevant, consider translating estimates of relative risk into absolute risk for a meaningful time period<br>**Tables 2, 3 and 4**
Other analyses	17	Report other analyses done—eg analyses of subgroups and interactions, and sensitivity analyses<br>**Results section, household cohort – difference-in-differences, household cohort coping strategies, and household cohort household expenditure sub-sections.**
**Discussion**		
Key results	18	Summarise key results with reference to study objectives<br>**Discussion section, summary of results sub-section, 1st paragraph.**
Limitations	19	Discuss limitations of the study, taking into account sources of potential bias or imprecision. Discuss both direction and magnitude of any potential bias<br>**Discussion section, strengths & limitations sub-section.**
Interpretation	20	Give a cautious overall interpretation of results considering objectives, limitations, multiplicity of analyses, results from similar studies, and other relevant evidence<br>**Discussion section, food security improvements, and the CBI did not appear to reduce the risk of acute malnutrition sub-sections.**
Generalisability	21	Discuss the generalisability (external validity) of the study results<br>**Discussion section, food security improvements, and the CBI did not appear to reduce the risk of acute malnutrition sub-sections.**
**Other information**		
Funding	22	Give the source of funding and the role of the funders for the present study and, if applicable, for the original study on which the present article is based<br>**See 'Financial Disclosure' section.**

*Give information separately for exposed and unexposed groups.
