# Supplementary material for: A cash-based intervention and the risk of acute malnutrition in children aged 6–59 months living in internally displaced persons camps in Mogadishu, Somalia: A non-randomised cluster trial
Source: PLoS Med. 2018 Oct 29;15(10):e1002684. doi: 10.1371/journal.pmed.1002684 (PMC6205571; doi:10.1371/journal.pmed.1002684)
Supplement: S1 Table — (PDF) [file pmed.1002684.s004.pdf]

S1 Table. Key validation studies and adaptation of tools.

Selected field and validation studies using the REFANI-S food security and dietary intake measures in low income and African contexts. Adapted from [1]

Tool	Location	Purpose	Measure used	Reference
**FCS**	Burundi, Haiti, Sri Lanka	Validation of the quantitative aspect (food quantity) of the FCS and alternative diet diversity and food frequency indicators constructed from FCS against calorie consumption per capita per day from survey data	• FCS (unmodified)	[2]
	Burkina Faso, Lao People's Democratic Republic, northern Uganda	Comparison of the performance of FCS and HDDS in food insecurity assessment	• FCS (unmodified)<br>• HDDS (unmodified)	[3]
**DDS**	South Africa	Description of the relationship between a DDS and other food security measures in a livelihood survey	• A household score (0–9) based on 9 food groups eaten in the previous 24h (modified HDDS)	[4]
	Bangladesh, Egypt, Ghana, India, Kenya, Malawi, Mali, Mexico, Mozambique, and The Philippines	Identification of the extent to which diet diversity is an appropriate measure of household food access	• In all countries but Bangladesh and Philippines, number of unique foods consumed in previous 7d (Bangladesh: previous 24h; Philippines: previous 1mo.)	[5]
**HFIAS**	Tanzania	Assessment of the validity and reliability of HFIAS among poor, rural households in Tanzania	• 9-item HFIAS with 30-day recall period	[6]
	Ethiopia	Assessment of the validity and dependability of HFIAS among community health volunteers	• 9-item HFIAS administered at 3-time points in 2008	[7]
**HHS & HFIAS**	Ethiopia	Examine household food security and hunger	• HHS and HFIAS	[8]
**rCSI**	Ghana, Kenya, Eritrea, Burundi, Ethiopia, Zimbabwe, Zambia, Malawi, Lesotho, Mozambique, Swaziland	Identify a sub-set of individual coping behaviours common to all surveys, whose severity is regarded as broadly similar by households across these studies	• CSI and rCSI	[9]
**MAHFP**	Bangladesh	Improve understanding of household food security in Bangladesh.	• MAHFP<br>• WDDS	[10]
	Blantyre (Malawi), Cape Town (South Africa), Gaborone (Botswana), Harare (Zimbabwe), Johannesburg (South Africa), Lusaka (Zambia), Maputo (Mozambique), Manzini (Swaziland), Maseru (Lesotho), Msunduzi (South Africa), and Windhoek (Namibia)	Understand the challenges of food access faced in poor urban neighbourhoods	• HFIAS<br>• HFIAP<br>• HDDS	[11]

**MUAC**	Zambia	Determine the sensitivity, specificity, and predictive value of arm circumference measurement as an indicator of nutritional status	• MUAC	[12]
	Ghana	Assess the inter- and intra-observer variability in MUAC measurements of under-five children by community health nurses in selected communities of the Northern Region in Ghana	• MUAC	[13]

FCS: Food Consumption Score. DDS: Dietary Diversity Score. HDDS: Household Dietary Diversity Score. WDDS: Women's Dietary Diversity Score. HFIAS: Household Food Insecurity Access Scale. HFIAP: The Household Food Insecurity Access Prevalence Indicator. HHS: Household Hunger Scale. rCSI: Reduced Coping Strategies index. MAHFP: Months of Adequate Household Food Provisioning. MUAC: Mid-Upper Arm Circumference.

Adaptation of tools to the local context

Tools were translated into the Somali language by a local translator. They were then back translated into English, and then during a two-weeks training session, the data collection team, who were bilingual English and Somali speakers, discussed the tools in detail and changes were undertaken, if needed, to make sure that tools were using locally available foods, terms and events, as per recommended practice in the different questionnaire guidelines. Following translation and adaptations, the data collection teams role-played and pretested the tools after which a final debriefing session and the final adaptations of the questionnaires were made, ensuring that the correct Somali terminologies were used.

References

1. Jones AD, Ngure FM, Pelto G, Young SL. What Are We Assessing When We Measure Food Security? A Compendium and Review of Current Metrics. Adv Nutr. 2013;4: 481–505. doi:10.3945/an.113.004119
2. Wiesmann D, Bassett L, Benson T, Hoddinott J. Validation of the World Food Programme's Food Consumption Score and Alternative Indicators of Household Food Security. IFPRI Discuss Pap. 2009;00870: 1–105.
3. Kennedy G, Berardo A, Papavero C, Horjus P, Ballard T, Dop M, et al. Proxy measures of household food consumption for food security assessment and surveillance: comparison of the household dietary diversity and food consumption scores. Public Health Nutr. 2010;13: 2010–2018. doi:10.1017/S136898001000145X
4. Faber M, Schwabe C, Drimie S. Dietary diversity in relation to other household food security indicators. Int J Food Safety, Nutr Public Heal. 2009;2: 1. doi:10.1504/IJFSNPH.2009.026915
5. Hoddinott J, Yohannes Y. Dietary Diveristy as a Food Security Indicator. Discussion Paper BRIEFS. Washington, D.C.; 2002.
6. Knueppel D, Demment M, Kaiser L. Validation of the Household Food Insecurity Access Scale in rural Tanzania. Public Health Nutr. 2010;13: 360. doi:10.1017/S1368980009991121
7. Maes KC, Hadley C, Tesfaye F, Shifferaw S, Tesfaye YA. Food Insecurity among Volunteer AIDS Caregivers in Addis Ababa, Ethiopia Was Highly Prevalent but Buffered from the 2008 Food Crisis. J Nutr. 2009;139: 1758–1764. doi:10.3945/jn.109.108548
8. Regassa N, Stoecker BJ. Household food insecurity and hunger among households in Sidama district, southern Ethiopia. Public Health Nutr. 2012;15: 1276–

1283. doi:10.1017/S1368980011003119
9. Maxwell D, Caldwell R, Langworthy M. Measuring food insecurity: Can an indicator based on localized coping behaviors be used to compare across contexts? Food Policy. Elsevier Ltd; 2008;33: 533–540. doi:10.1016/j.foodpol.2008.02.004
10. Harris-Fry H, Azad K, Kuddus A, Shaha S, Nahar B, Hossen M, et al. Socio-economic determinants of household food security and women's dietary diversity in rural Bangladesh: a cross-sectional study. J Heal Popul Nutr. Journal of Health, Population and Nutrition; 2015;33: 2. doi:10.1186/s41043-015-0022-0
11. Crush J, Frayne B, Pendleton W. The Crisis of Food Insecurity in African Cities. J Hunger Environ Nutr. 2012;7: 271–292. doi:10.1080/19320248.2012.702448
12. McDowell I, King FS. Interpretation of arm circumference as an indicator of nutritional status. Arch Dis Child. 1982;57: 292–296. doi:10.1136/adc.57.4.292
13. Saeed H, Mogendi J, Akparibo R, Kolsteren P. Reliability of Mid-Upper Arm Circumference Measurements taken by Community Health Nurses. Curr Res Nutr Food Sci J. 2015;3: 26–35. doi:10.12944/CRNFSJ.3.1.04
