# Supplementary material for: A cash-based intervention and the risk of acute malnutrition in children aged 6–59 months living in internally displaced persons camps in Mogadishu, Somalia: A non-randomised cluster trial
Source: PLoS Med. 2018 Oct 29;15(10):e1002684. doi: 10.1371/journal.pmed.1002684 (PMC6205571; doi:10.1371/journal.pmed.1002684)
Supplement: S2 Table — (PDF) [file pmed.1002684.s005.pdf]

S2 Table. Baseline coping strategies of the household cohort.

Coping Strategy	Control[1]		Intervention[1]		Intervention minus Control	
	Mean	95% CI	Mean	95% CI	Difference	95% CI
Number of days using strategy/7 days						
Relied on less preferred and less expensive foods	5.98	5.38; 6.59	3.83	3.55; 4.11	-2.15**	-2.82; -1.49
Borrowed food or relied on help from friends/family	3.33	2.77; 3.89	3.00	2.52; 3.48	-0.33	-1.07; 0.40
Reduced number of meals eaten in a day	3.17	2.74; 3.60	3.16	2.76; 3.56	-0.01	-0.60; 0.58
Limited portion size at mealtimes	3.52	3.18; 3.86	3.21	2.79; 3.62	-0.31	-0.85; 0.22
Restricted consumption by adults in order for small children to eat	2.39	1.78; 3.00	2.51	1.99; 3.04	0.12	-0.69; 0.93
Percent of household using strategy/7 days						
Relied on less preferred and less expensive foods	100	96.8; 100	100	96.7; 100	0.00	-3.18; 3.35
Borrowed food or relied on help from friends/family	91.5	80.5; 96.5	87.4	76.0; 93.8	-4.07	-15.4; 7.31
Reduced number of meals eaten in a day	96.6	89.8; 98.9	98.2	87.2; 99.8	1.62	-3.70; 6.94
Limited portion size at mealtimes	97.4	93.0; 99.1	99.1	93.4; 99.9	1.66	-1.55; 4.87
Restricted consumption by adults in order for small children to eat	81.2	63.0; 91.7	93.7	85.5; 97.4	12.5	-2.75; 27.7

[1] Control n = 117, intervention n = 111.

*p < 0.05; **p < 0.01
