# Supplementary material for: A cash-based intervention and the risk of acute malnutrition in children aged 6–59 months living in internally displaced persons camps in Mogadishu, Somalia: A non-randomised cluster trial
Source: PLoS Med. 2018 Oct 29;15(10):e1002684. doi: 10.1371/journal.pmed.1002684 (PMC6205571; doi:10.1371/journal.pmed.1002684)
Supplement: S3 Table — (PDF) [file pmed.1002684.s006.pdf]

S3 Table. Baseline 30-day expenditure of the household cohort (in US dollars).

Item	Control		Intervention		Intervention minus Control	
	**Mean**	**95% CI**	**Mean**	**95% CI**	**Difference**	**95% CI**
Food						
Cereals	16.6	12.4; 20.8	22.4	11.3; 33.5	5.81	-6.06; 17.7
Roots & Tubers	1.74	0.81; 2.68	2.89	1.52; 4.27	1.15	-0.51; 2.81
Pulses, Beans & Nuts	2.97	2.12; 3.82	3.76	2.10; 5.41	0.78	-1.08; 2.64
Vegetables	3.85	2.95; 4.76	5.17	3.06; 7.28	1.32	-0.98; 3.61
Fruits	2.51	1.92; 3.11	3.65	2.51; 4.78	1.14	-0.15; 2.42
Meat, Fish & Eggs	4.03	3.31; 4.74	8.40	6.99; 9.80	4.37**	2.79; 5.95
Dairy Products	5.16	3.77; 6.49	6.84	6.00; 7.67	1.71*	0.12; 3.30
Fats & Oils	4.42	3.55; 5.29	6.38	5.77; 6.99	1.96**	0.90; 3.02
Sugary Products	5.83	4.63; 7.02	7.55	6.40; 8.70	1.72*	0.06; 3.38
Condiments	1.94	1.48; 2.40	1.76	1.26; 2.26	-0.18	-0.86; 0.50
Total food expenditure	49.0	38.1; 59.9	68.8	51.7; 85.9	19.8	-0.51; 40.0
Non-food						
Firewood/cooking fuel	6.21	4.86; 7.56	6.17	4.93; 7.41	-0.04	-1.88; 1.79
Cigarettes, tobacco, khat	0.46	-0.05; 0.97	1.02	-0.55; 2.59	0.56	-1.06; 2.21
Drinking water	5.17	4.02; 6.33	6.26	5.28; 7.24	1.09	-0.43; 2.61
Education	2.67	1.65; 3.68	1.54	0.48; 2.60	-1.12	-2.59; 0.34
Health	2.84	2.33; 3.35	2.11	0.95; 3.27	-0.72	-1.99; 0.54
Clothing	2.07	0.61; 3.53	1.28	0.47; 2.08	-0.80	-2.46; 0.87
Transport	4.30	3.61; 5.00	2.63	1.43; 3.83	-1.67*	-3.06; -0.28
Debt repayment	0.88	0.41; 1.36	1.12	0.59; 1.64	0.23	-0.48; 0.94
Sending remittances	0.11	-0.10; 0.32	0.24	-0.21; 0.69	0.13	-0.36; 0.63
House or buildings	1.72	-0.10; 3.54	0.80	0.26; 1.34	-0.92	-2.82; 0.98
Shop or trading facilities	0.10	-0.10; 0.29	0.00	0.00; 0.03	-0.10	-0.29; 0.10
Purchasing land	0.00	0.00; 0.03	0.00	0.00; 0.03	0.00	-0.03; 0.03
Farming items	0.00	0.00; 0.03	0.01	-0.1; 0.02	0.01	-0.01; 0.02
Livestock	0.00	0.00; 0.03	0.00	0.00; 0.03	0.00	-0.03; 0.03
Celebrations	0.14	0.02; 0.26	0.14	-0.02; 0.29	-0.01	-0.20; 0.19
Total non-food expenditure	26.9	20.8; 32.4	23.1	16.5; 29.7	-3.46	-12.2; 5.32

[1] Control n = 117, intervention n = 111.

*$p < 0.05$; **$p < 0.01$
