# Supplementary material for: A cash-based intervention and the risk of acute malnutrition in children aged 6–59 months living in internally displaced persons camps in Mogadishu, Somalia: A non-randomised cluster trial
Source: PLoS Med. 2018 Oct 29;15(10):e1002684. doi: 10.1371/journal.pmed.1002684 (PMC6205571; doi:10.1371/journal.pmed.1002684)
Supplement: S4 Table — (PDF) [file pmed.1002684.s007.pdf]

**S4 Table.** Baseline differences in indicators associated with the assessment of vulnerability and targeting of the intervention.

| Vulnerability Criteria | Related study indicators | Data source | | Significantly different at baseline | Notes |
|---|---|---|---|---|---|
| | | Household cohort | Child cohort | | |
| Households headed by elderly, children, women, or people with disabilities | Female household head (Table 1) | Yes | No | No | Intervention clusters were more female headed (10% greater) |
| Orphans in the household | No data collected in the study | -- | -- | -- | -- |
| All household members had no income sources | Household income last 30 days (Table 1) | Yes | No | **Yes** | Intervention clusters had greater income (US $26 more income) |
| | Household expenditure last 30 days (Table1) | Yes | No | No | Intervention clusters had greater expenditure (US $16 more expenditure) |
| Households had children recently recovered from SAM | Child mean WHZ (Table 1) | Yes | No | No | -- |
| | Child mean MUAC (Table 1 and S5 Table) | Yes | Yes | No | -- |
| | Prevalence of acute malnutrition by low WHZ (Table 1) | Yes | No | No | -- |
| | Prevalence of acute malnutrition by low MUAC (Table 1) | Yes | No | No | -- |
| Households had high dependency ratios | Household total dependency ratio (Table 1) | Yes | No | **Yes** | Intervention clusters had lower dependency ratios |
| | Household child dependency ratio (Table 1) | Yes | No | **Yes** | Intervention clusters had lower dependency ratios |
| | Household aged dependency ratio (Table 1) | Yes | No | No | -- |
| The presence of pregnant and/or lactating women | Women reported being pregnant (Table 1) | Yes | No | No | -- |
| Recent eviction | No data collected in the study | -- | -- | -- | All intervention clusters were recently evicted |
