# Supplementary material for: A cash-based intervention and the risk of acute malnutrition in children aged 6–59 months living in internally displaced persons camps in Mogadishu, Somalia: A non-randomised cluster trial
Source: PLoS Med. 2018 Oct 29;15(10):e1002684. doi: 10.1371/journal.pmed.1002684 (PMC6205571; doi:10.1371/journal.pmed.1002684)
Supplement: S5 Table — (PDF) [file pmed.1002684.s008.pdf]

S5 Table. Linear and ordered logistic regression on endline Child Dietary Diversity Score.

Variable	Linear regression				Ordered logistic regression[1]			
	Unadjusted		Adjusted		Unadjusted		Adjusted	
	β-coefficient	95% CI	β-coefficient	95% CI	β-coefficient	95% CI	β-coefficient	95% CI
Time (endline)	0.49**	0.25; 0.72	0.49**	0.25; 0.73	0.77**	0.39; 1.15	0.78**	0.37; 1.18
Arm (intervention)	0.58**	0.26; 0.90	0.38*	0.00; 0.75	0.92**	0.40; 1.43	0.58	-0.01; 1.17
Difference-in-differences (time x arm)	0.62*	0.09; 1.16	0.62*	0.08; 1.15	1.06*	0.14; 1.98	1.11*	0.15; 2.07
Baseline age dependency ratio			-0.03	-0.11; 0.06			-0.04	-0.17; 0.09
Baseline reduced coping strategies index			0.00	-0.01; 0.01			0.00	-0.02; 0.03
Baseline household income in US $			0.01**	0.00; 0.01			0.01**	0.01; 0.02
Child's age in months			0.00	-0.01; 0.01			0.00	-0.01; 0.01

[1]Coefficients are interpreted as the log odds of having a one-unit higher Child Dietary Diversity Score.

*$p < 0.05$; **$p < 0.01$
