# Supplementary material for: A cash-based intervention and the risk of acute malnutrition in children aged 6–59 months living in internally displaced persons camps in Mogadishu, Somalia: A non-randomised cluster trial
Source: PLoS Med. 2018 Oct 29;15(10):e1002684. doi: 10.1371/journal.pmed.1002684 (PMC6205571; doi:10.1371/journal.pmed.1002684)
Supplement: S6 Table — (PDF) [file pmed.1002684.s009.pdf]

S6 Table. Sample characteristics and MUAC at each surveillance time point.

Time point	Control					Intervention				
	Sample	Age (months)	Female	MUAC (cm)	MUAC <12.5 cm	Sample	Age (months)	Female	MUAC (cm)	MUAC <12.5 cm
	n	Mean ± S.D.	Percent	Mean ± S.D.	Percent (95% CI)	*n*	Mean ± S.D.	Percent	Mean ± S.D.	Percent (95% CI)
June [1]	1,135	35.5 (13.7)	51.5	14.6 (1.14)	-	697	37.0 (14.1)	48.5	14.5 (1.07)	-
July	1,126	36.3 (13.9)	52.4	14.7 (1.19)	1.9 (1.16, 2.84)	698	38.0 (14.1)	47.6	14.7 (1.10)	2.2 (1.21,3.52)
August	1,171	37.4 (13.9)	52.6	14.8 (1.17)	1.7 (1.05, 2.63)	696	38.5 (14.1)	47.4	14.7 (1.11)	1.3 (0.59, 2.44)
September	1,132	38.6 (13.9)	52.1	14.8 (1.15)	1.7 (1.01, 2.61)	672	39.7 (14.1)	47.9	14.7 (1.11)	1.6 (0.82, 2.91)
October	1,112	38.8 (14.1)	52.5	14.9 (1.19)	1.9 (1.17, 2.87)	656	40.9 (14.0)	47.5	14.8 (1.07)	1.7 (0.84, 2.98)

MUAC: Mid-Upper Arm Circumference

[1] The were no significant differences in mean age or mean MUAC between the study arms at baseline.
