# Supplementary material for: A cash-based intervention and the risk of acute malnutrition in children aged 6–59 months living in internally displaced persons camps in Mogadishu, Somalia: A non-randomised cluster trial
Source: PLoS Med. 2018 Oct 29;15(10):e1002684. doi: 10.1371/journal.pmed.1002684 (PMC6205571; doi:10.1371/journal.pmed.1002684)
Supplement: S7 Table — The intervention sample includes children whose mothers or primary carers were confirmed cash recipients. (PDF) [file pmed.1002684.s010.pdf]

S7 Table. Effect of the intervention on time to first episode of acute malnutrition or death. The intervention sample only includes children whose mothers or primary carers were confirmed cash recipients.

**Variable**	**Control**	**Intervention**	***p*-Value**
Sample, *n*	1,379	465	
Child-months observed, *n*	5,632	2,113	
Acute malnutrition by low MUAC cases, *n*	52	16	
Oedema cases, *n*	0	0	
Death, *n*	11	1	
Acute malnutrition incidence rate, cases/100 child-months	0.92 (0.53; 1.14)	0.76 (0.46; 1.24)	
Mortality rate, deaths/100 child-months	0.20 (0.11; 0.35)	0.05 (0.01; 0.34)	
Acute malnutrition incidence hazard ratio	Reference	0.81 (0.39; 1.73)	0.6
Adjusted[1] acute malnutrition incidence hazard ratio	Reference	0.86 (0.38; 1.98)	0.7
Mortality hazard ratio	Reference	0.25 (0.05; 1.30)	0.09

Acute malnutrition by low MUAC was defined as MUAC <12.5cm and/or oedema. MUAC: Mid-upper arm circumference. Values in parenthesis are 95% CIs.
[1]Adjusted for age (in months) and male sex.
